# Innovative Epicardial Bigels Containing Amiodarone Hydrochloride: Pharmacotechnical and Analytical Characterization

**DOI:** 10.3390/ph17111511

**Published:** 2024-11-09

**Authors:** Cezara Pintea, Robert-Alexandru Vlad, Paula Antonoaea, Emőke Margit Rédai, Magdalena Bîrsan, Enikő-Csilla Barabás, Andrei Manea, Iulia Alexandra Pușcaș, Adriana Ciurba

**Affiliations:** 1 Pharmaceutical Technology and Cosmetology Department, Faculty of Pharmacy, George Emil Palade University of Medicine, Pharmacy, Science, and Technology of Targu Mures, 540142 Targu Mures, Romania; 2 Medicine and Pharmacy Doctoral School, George Emil Palade University of Medicine, Pharmacy, Science, and Technology of Targu Mures, 540142 Targu Mures, Romania; 3 Department of Drug Industry and Pharmaceutical Biotechnology, “Grigore T. Popa” University of Medicine and Pharmacy from Iasi, 700115 Iasi, Romania; 4 Department of Laboratory Medicine, Mures, County Hospital, 540136 Targu Mures, Romania; 5 Department of Radiology, Mures, County Emergency Hospital, 540136 Targu Mures, Romania; 6 The Department of Cardiovascular Surgery, Emergency Institute for Cardiovascular Diseases and Transplantation Targu Mures, 540142 Targu Mures, Romania

**Keywords:** amiodarone hydrochloride, bigels, texture analysis, rheological evaluation, content assay, microbiological evaluation

## Abstract

Background/Objectives: The search for novel ways of providing treatment also targets the development of formulations used in drug delivery. Among the important characteristics of pharmaceutical gels are their ability to penetrate membranes, their capability to offer rapid response, and their capacity to avoid the hepatic metabolization route followed by many drugs. Bigels combine the advantages of both hydrogels and oleogels, creating a biphasic system that might improve the solubility of amiodarone in water, which is otherwise poorly soluble. This study aimed to succeed in formulating stable amiodarone hydrochloride bigels (coded from ABG1-ABG6) destined for atrial application and evaluating them from a pharmacotechnical perspective. Methods: Three of the six initial formulations presented stability and underwent studies of spreadability, rheology, drug content, textural properties, and microbiological activity. A statistical analysis was performed on penetrometry and drug assay data. Results: The spreadability varied from 1734.07 mm^2^ (ABG1) to 2163.85 mm^2^ (ABG6), while the drug concentration ranged between 1.35 and 1.49% (*w*/*w*). The textural profile analysis highlighted superior hardness, cohesiveness, and resilience for ABG6 and higher adhesion for ABG2. Both presented pseudoplastic thixotropic behavior, while a plastic thixotropic flow was registered in the case of ABG1. Conclusions: All three bigels are suitable for amiodarone incorporation; however, the influence of the type of ingredients chosen on the texture and properties of the formulations was reflected in the data gathered upon evaluation.

## 1. Introduction

The current tendencies of scientific research in the pharmaceutical field provide new insights into the novel discoveries of never-before-used active substances, as well as innovative ways of transportation and formulation. Due to the high costs of manufacturing new molecules, more and more attention has been directed to reinventing ways for the administration of already-studied drugs. In this case, the local usage of amiodarone (AMDR) was proposed for a potential decrease in the appearance of postoperative atrial fibrillation by developing bigel formulations.

**Figure 1:**
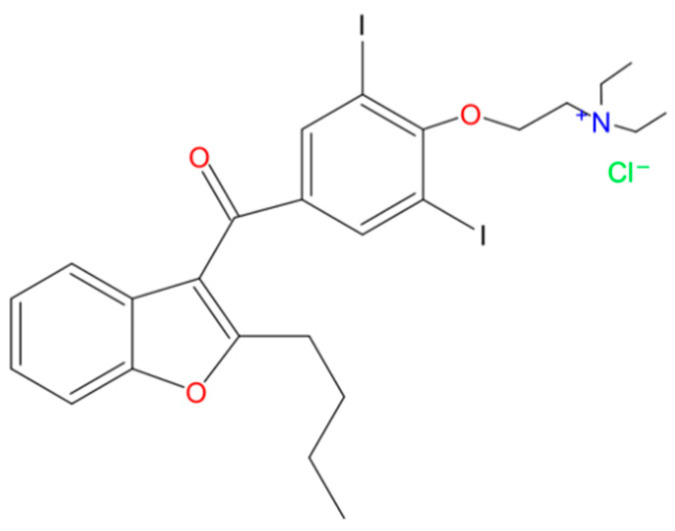
The chemical structure of amiodarone hydrochloride.

Emerging from a 1962 search for coronary dilatators, in Belgium, many benzofuran derivatives were synthesized, among which benzarone and benziodarone preceded the appearance of amiodarone, which manifested antianginal and antiarrhythmic properties [1,2]. The latter compound contains two iodine atoms, as illustrated in Figure 1.

Amiodarone is often used in the form of hydrochloride salt and can be found under the International Union of Pure and Applied Chemistry (IUPAC) name of (2-butylbenzofuran-3-yl)[4-[2-(diethylamino)ethoxy]-3,5-diiodophenyl] methanone hydrochloride. Found in the form of a white crystalline powder, the drug’s solubility in water is within a small percent, as opposed to being soluble in methylene chloride, methanol, and polysorbate 80 [3,4]. Given that AMDR presents high permeability through the intestinal membrane and low aqueous solubility, it belongs to the second class of the Biopharmaceutical Classification System (BCS) [5]. Due to its lipophilic nature and high degree of plasma protein binding, the substance exhibits an affinity for adipose tissue and many organs, having a substantial volume of distribution. Regarding metabolism, the primary pathway through the liver is the cytochrome P450 oxidative enzyme system, resulting in *N*-desethylamiodarone as the main metabolite [6,7].

As an active pharmaceutical ingredient (API), amiodarone is a class III antiarrhythmic globally prescribed for treating various cardiac rhythm disorders, focusing on supraventricular or ventricular arrhythmias and atrial fibrillation [8]. 

According to the literature, the risk of developing postoperative atrial fibrillation (POAF) following open-heart procedures stretches from 15% to 50%, depending on the intervention type [9,10]. The antiarrhythmic effect has been associated with reducing the incidence of POAF. In many cases, initiating AMDR therapy before elective cardiac surgery proved efficient regardless of the route of administration, oral or intravenous [11,12]. From this belief, the search for a form of localized drug delivery was initiated to avoid negative effects following the metabolism of AMDR. Local application may result in lower levels of amiodarone in the bloodstream, reducing the likelihood of long-term organ toxicity. Researchers have developed formulations and innovative means of transport varying from the long-term catheter-based intrapericardial infusion of amiodarone solution [13,14] to a biodegradable, cross-linkable dextran disc with AMDR [15] or an amiodarone-eluting epicardial bilayered patch [16] having highly complex development technologies and means of administration. However, many opted to incorporate the API in hydrogels utilizing various excipients and delivery methods, observed through animal testing and clinical trials. For example, Garcia et al. used a pig and rodent model to investigate the drug’s delivery technique of creating a temporary compartment for applying the hydrogel on the heart’s surface [17]. Teymuraz Kanametov and Vladimir Shvartz introduced another form of biopolymer based on sodium alginate for developing a hydrogel material called “Colegel”, whose efficacy was tested on 46 rabbits [18,19]. Bolderman et al. explored the epicardial application of an AMDR hydrogel consisting of a surgical sealant (CoSeal^®®^) with PEG polymers on goats [20]. A similar approach using the same matrix for the development of a sprayable formula for diffuse application was described by Feng et al. in a promising prospective study carried out on 100 patients [21]. Another prospective study conducted by Wang et al. on 150 patients compared the benefits of a sprayable hydrogel formula with amiodarone and triamcinolone next to a control group. The clinical study showed a significant reduction in POAF incidence for the biatrial epicardial delivery of amiodarone-releasing hydrogel [22]. Local amiodarone application was later studied by Greenstein et al. on three series of patients treated with either no active substance, an AMDR topical hydrogel, or an amiodarone-soaked sealant patch, summing 200 patients. However, this retrospective study presented no clear benefits supporting the degression of the atrial fibrillation incidence rate within the study group [23]. Many of these findings highlighted lower amiodarone concentrations in the blood or other organs but successful delivery to the atrial tissue. Thus, the promising results suggested continuing research.

Gels are elastic colloidal materials that distinguish themselves through their unique tri-dimensional structure and the stability presented in the application area. Due to the broad spectrum of applicability, they are used in many industries besides the medical and pharmacological domains [24]. The key advantage provided lies in the capacity to cross membranes and deliver a fast local effect, avoiding the first-pass metabolization route and possible drug interactions or adverse reactions [25].

One of the biggest limitations of using amiodarone is the drug’s lack of solubility, which inspired the use of a formulation combining hydrophilic and lipophilic gels. For the stability of the bigel, emulsifiers were also added to the mixture. Hydrogel (HGL) is a polymeric cross-linked material with a three-dimensional configuration that can absorb large quantities of water, swelling but not dissolving. This behavior grants them features such as mechanical, shape-shifting, and elastic properties depending on the network density and physicochemical interactions [26,27]. The form of the gel is influenced by the quantity of the polymer or its viscosity. When increasing the two, firmer gels can be obtained. They have been produced using either natural (alginate, collagen, guar gum) or synthetic polymers (polyethylene glycol, polyacrylic acid, polyvinyl pyrrolidone), dispersed over aqueous solutions. For topical preparations, cellulose-based polymers have been widely used [28,29]. Owing to their porous system which facilitates the loading of different active substances, hydrogels are widely used for diagnostic, therapeutic, or surgical purposes [30,31]. Oleogels are newer discoveries, originally developed to replace food additives for reducing the consumption of trans and saturated fatty acids [32]. Their preparation requires heating the solvent before combining it with the oleogelator. The liquid phase, represented by a mineral or a vegetal oil, is immobilized within the network upon cooling [33]. The gelation process is caused by two types of oleogelators whose purpose is to form bonds entrapping the oil. Low-molecular-weight oleogelators (LMOGs) are self-assembling molecules that create weak physical bonds and reunite classes of waxes, fatty acids, phytosterols, and ceramides. High-molecular-weight oleogelators (HMOGs) form three-dimensional oily networks through hydrogen bonding and are represented by proteins and polysaccharides. Among these, waxes such as Carnauba wax, Beeswax, and *Candelilla* wax are the most used and usually melt while oil heating [34,35].

Due to their oily constitution, oleogels can also function as transporters for lipophilic biotherapeutic agents [34,36]. Combining the two semisolid bases and reuniting their properties, an innovative formulation originated as a bigel (BGL). Its authenticity comes from the ability to deliver both hydrophilic and hydrophobic drugs due to the unity of two gelled phases, unlike emulsions or creams [37,38]. In this regard, Martinez et al. provided a comparison between bigels and emulsions with Vitamin E [39]. To capitalize on this diversity, their use in drug delivery is expanding rapidly. Drugs from various categories are being considered for the transdermal application of bigels, including antibiotics, antifungals, anti-inflammatories, and other active agents. For example, a Polish study related the successful formulation of bigels with ketoconazole [40]. Their good spreadability, emollient, and hydrating qualities enhance the API’s skin penetration; thus, research continues to expand BGL’s use for buccal and vaginal delivery. An encouraging discovery in this area can be found in developing mucoadhesive bigels containing tenofovir and maraviroc designed to prevent HIV transmission [41]. According to this, there has been an increased interest in bigels in the cosmetic, pharmaceutic, and food industries [42,43,44,45].

Another important pharmaceutical formulation that can be administered locally is the transdermal patch, which still has some disadvantages compared to semisolid formulations (bigels):

- The development and optimization of patches are time-consuming in comparison to the selected semisolid preparations.
- In the case of bigels, both hydrophilic and lipophilic active ingredients can be incorporated (a broader spectrum of ingredients can be incorporated).
- Bigels are easier to manipulate in comparison to patches, which in some cases are visible and occupy a large volume.
- The risk of local side effects (itchiness, rash) is reduced in the case of bigels compared to patches [42,43,44,45,46,47].

The focal point of this study lies in developing pharmaceutical products with amiodarone hydrochloride proposed for local heart delivery, which has reduced off-target organ toxicity and purposely downscales the incidence of POAF. A semisolid, biphasic matrix was considered for AMDR incorporation—a bigel containing both a hydrophilic and a lipophilic part—considering that amiodarone hydrochloride is not soluble in hydrophilic components but is soluble in the lipophilic matrix, the selected pharmaceutical formulation comprising both the properties of a hydrogel and the ones that are characteristic for an oleogel (organogel).

## 2. Results and Discussion

### 2.1. Preformulation

To obtain stable bigel preparations, the formulation process suffered alterations constantly. For the first attempt, the Amiodarone Hydrochloride 50 mg/mL Concentrate for Solution for Injection/Infusion (Hameln Pharma, Hameln, Germany) was used in the hydrogel part. However, the results were unsatisfactory due to the uneven distribution and agglomeration of the active substance. Regarding the hydrogels, the preparation started with using xanthan gum (Ellemental, Oradea, Romania, origin: Germany) which proved ineffective in gelling. The solution reached was incorporating powder amiodarone hydrochloride into the formulation after the gelling process. The development of AMDR hydrogels was proposed as a possible formulation, but whilst developing this type of formulation, it was noticed that separation might occur, which included the lack of homogeneity. The development of AMDR-OGLs was not considered since the viscosity of the formed lipophilic system was reduced, and considering that this study aimed to obtain AMDR semisolid formulations with higher consistency, these matrixes were not suitable.

### 2.2. Macroscopic Evaluation and Stability

**Figure 2:**
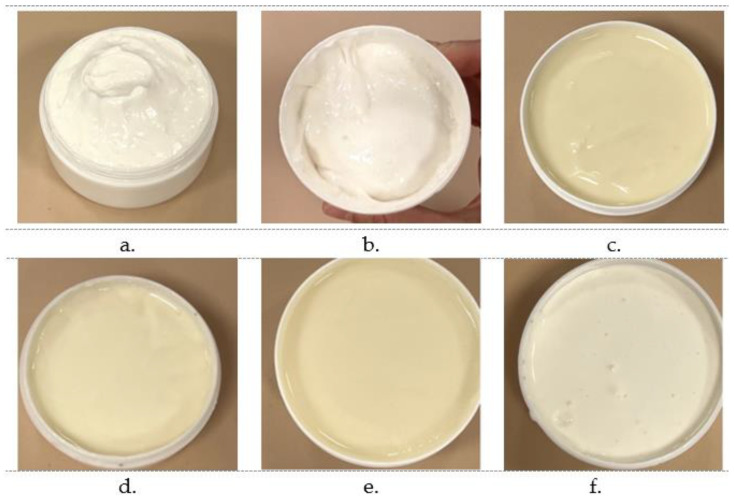
The appearance of each formulation of AMDR BGL immediately after preparation ((**a**) ABG1; (**b**) ABG2; (**c**) ABG3; (**d**) ABG4; (**e**) ABG5; (**f**) ABG6).

**Figure 3:**
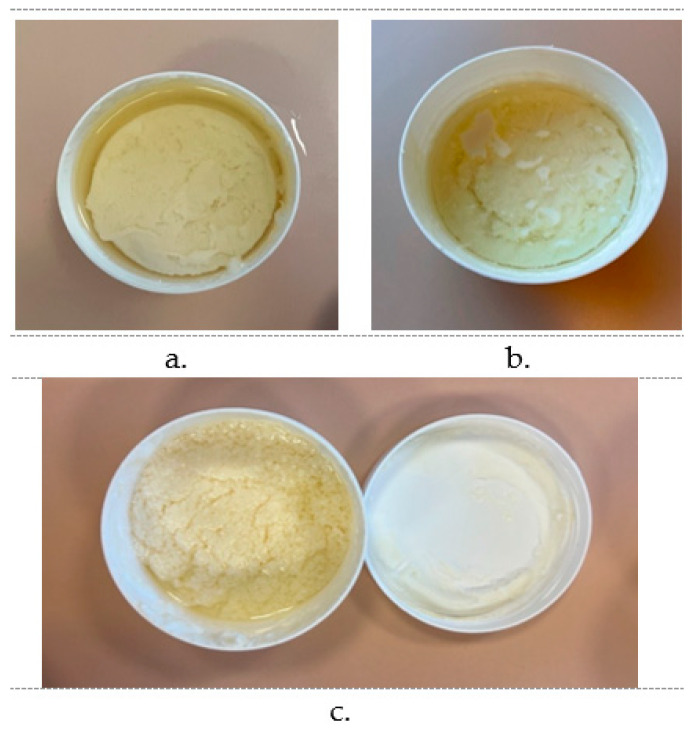
Unstable formulations ((**a**) ABG3, (**b**) ABG5, (**c**) ABG4).

**Figure 4:**
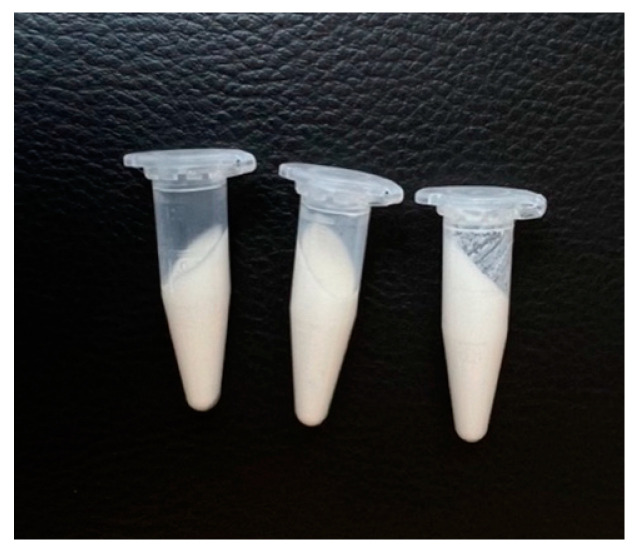
Bigel samples after centrifugation (from left to right: ABG1, ABG2, and ABG6).

Six formulations were made following the same preparation process and were subjected to pharmacotechnical evaluation. The initial consistency was homogenous and cream-like for all preparations and slightly firmer for ABG1 and ABG2, as shown in Figure 2. Following the stability evaluation over time, formulations ABG3 and ABG5 were the first to present separation with significant oil resurfacing, as presented in Figure 3. The excess oil was measured, proving that 25 g for ABG3 and 30 g for ABG5 of the lipophilic phase was separated from 100 g of bigel. After a longer period (+1 week), ABG4 presented uneven texture and quality alteration. Provided that the last gel’s separation was in a smaller proportion, Carbopol 940 proved to be a better gelling agent for the bigels where OGL Carnauba was used. Consequently, all three formulations were excluded from this study due to their lack of stability. On the other hand, the centrifugation applied for the remaining three formulations (21 days after the preparation) showed that they were stable. After 5 min of centrifugation at a speed of 3000 rpm, no separation was visible (Figure 4).

### 2.3. Spreadability Capacity

**Figure 5:**
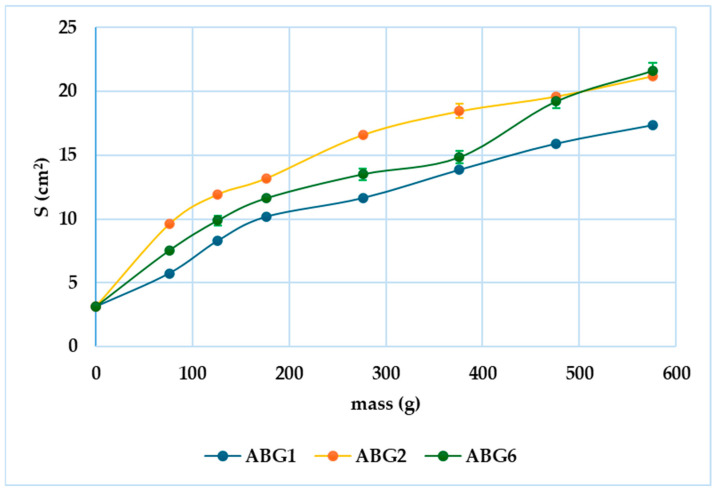
A spreadability graph for the three bigel formulations.

It is widely known that the spreadability of semisolid formulations is directly proportional to the increase in the weight applied. The surface areas of the gel spread variations were illustrated graphically (Figure 5).

As a result, the lowest spreadability capacity at the maximum weight applied was registered for ABG1 (17.34 cm^2^), while ABG2 and ABG6 reached similar values for the surface area (21.22 cm^2^ and 21.63 cm^2^, respectively). The results were also in agreement with the consistency of the bigels where the firmness decreased from ABG1 to ABG6, the latter one also presenting the highest spreading area. In addition, the strength of the correlation between the weight and the area was also evaluated using the statistic Pearson r test. Thus, the strongest correlation was recorded for ABG6, as shown in Table 1.

According to the classification made by Lardy [48], in the framework of experimental studies on different types of hydrogels, all formulations fall into the class of very stiff gels. Consequently, the circle diameter measured one minute after the application of the top plate is considered, which had values below 40 mm for all gels (ABG1—27 mm; ABG2—35 mm; ABG6—31 mm). Considering that the healthcare provider might be interested in a bigel that does not spread and does not migrate to other parts of the heart, ABG1 might be the most suitable formulation for this case scenario.

### 2.4. Rheology Evaluation

**Figure 6:**
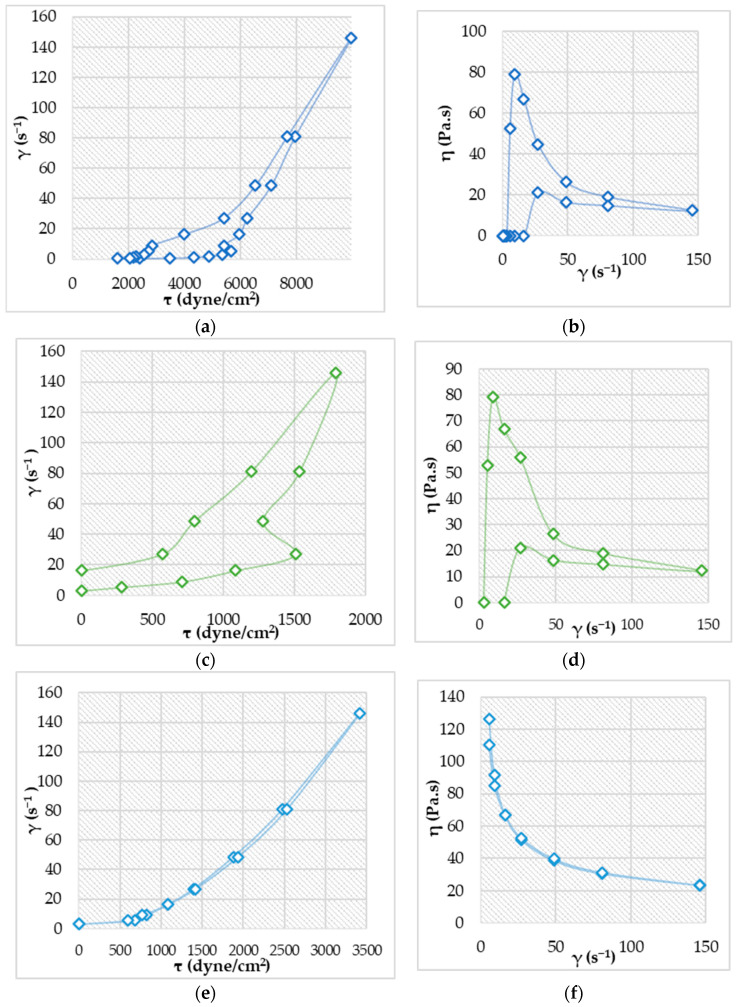
Flow and viscosity curves (**a**) flow curve for ABG1, (**b**) viscosity curve for ABG1, (**c**) flow curve for ABG2, (**d**) viscosity curve for ABG2, (**e**) flow curve for ABG6, (**f**) viscosity curve for ABG6.

Regarding the rheological behavior of the bigels, the analysis provided particularly distinctive values when calculating the shear stress (τ) and dynamic viscosity (η). The only formulation that used both viscosimeter sensitivity levels was ABG1, whereas for the other two, values were registered starting only with the sixth shear rate, a fact that can be explained that in this case, the CMC HGL was used in the highest concentration (5% *w*/*w*); this part of the matrix contributed to the higher viscosities. According to the data obtained, all three formulations were characterized by a thixotropic rheological behavior marked by two phases: the initial one, the destructuring, involves an increase in shear stress, causing the viscosity to decrease, while in the second phase of restructuring, the two parameters were reversed so that with the decrease in the shear stress, the viscosity increases. Different stress values are obtained on the two branches at the same shear rate, between which a hysteresis loop appears. The generated rheograms (Figure 6) suggest a plastic thixotropic flow for ABG1 and a pseudoplastic thixotropic for ABG2 and ABG6. For ABG2, phase separation was visible due to the pressure exerted by the spindle and the impact of the other forces which led to the destabilization of the gel matrix which can be correlated with the fact that in this case, HGL CBP 1% was used, while in the other two cases, the HGL CMC in the previously mentioned concentration was utilized, outlining that the bigel system containing CMC hydrogel and OGL Beeswax tends to be more stable from a rheological point of view.

All three bigels could be candidates for local application considering that all three exhibited good results regarding the viscosity. If a physician needs a formulation that is more viscous than the ABG1 formulation, it might be more useful, considering that the gel should not spread to other areas of the heart, remaining in a delimited region [49,50].

The concentration and ratio of the emulsifiers, and the ratio between the hydrophilic and lipophilic parts, influence the stability of the product. In the case of ABG2, this evaluation might also represent a test from which it can be concluded that at an increased number of rotations, the system tends to separate due to the high shear forces and the different components of the bigels [51,52,53].

### 2.5. Drug Content Evaluation

**Figure 7:**
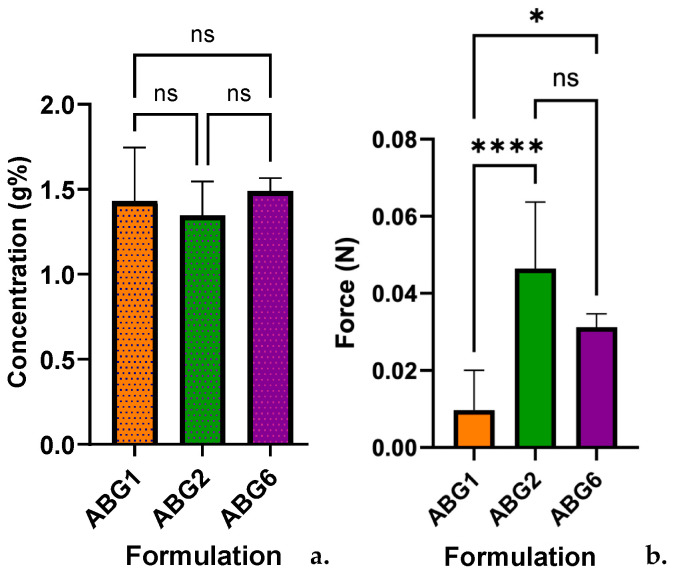
(**a**) Statistical comparison between gels using Brown–Forsythe and Welch’s ANOVA test, *p* > 0.05 (ns). (**b**) Statistical comparison between gels using the Kruskal–Wallis test, *p* > 0.05 (ns), *p* ≤ 0.05 (*), *p* ≤ 0.0001 (****).

The spectrophotometric determination of amiodarone concentration in each formulation used two wavelengths (λ), 206.9 nm for ABG1 and ABG2 and 242 nm for ABG6, to achieve selectivity in each of the three bigels evaluated. The concentration varied between 1.35 and 1.49 g% and fell within the limits established by the European Pharmacopoeia, 9th Edition [54], as shown in Table 2. By analyzing the data sets, from a statistical perspective, no statistically significant results were reported when comparing the indicators of central tendency, after performing the ANOVA statistical test of the Brown–Forsythe test (Figure 7a).

### 2.6. Texture Profile Analysis

The values obtained following the penetrometric determination are illustrated in Table 3 and highlight the gradual increase in the penetration distance (D) with a similar rate in all samples. The resemblance between formulations is striking; however, a bigger value for the maximum distance was registered for ABG1 (9.989 mm). The statistical analysis of the applied forces confirmed the existence of a strong statistically significant difference between ABG1 and ABG2 and a smaller one for the ABG1 and ABG6 data pair (Figure 7b). For this, the Kruskal–Wallis test for non-parametric distributions was used.

**Figure 8:**
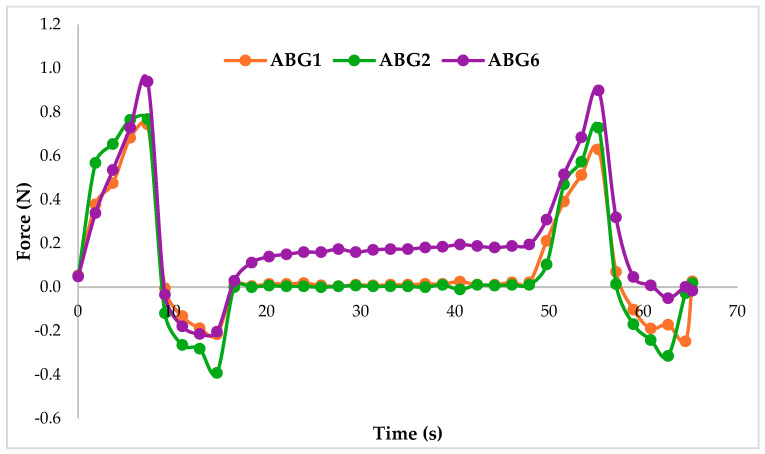
A graphical representation of the TPA compression cycles.

Additionally, the Texture Profile Analysis (TPA) registered 37 sets of values during the two cycles represented graphically (Figure 8), which provided quantifiable information in the form of numerical values about important characteristics of the formulations (Table 4). The highest value for the peak force of the first cycle was registered for formulation no. 6, granting it the highest hardness. Meanwhile, the lowest force was presented by ABG2, making it the most adhesive bigel of the three. In conformity with the hardness, ABG6 generated a superior value for the resisting deformation force and the highest internal cohesion, which reflects the strength of the internal bonds of the formulation’s network. Nevertheless, the elasticity measured showed no notable variations.

An important parameter that might be useful for a physician who is applying this epicardial gel is the adhesion force; from this point of view, ABG6 has the highest value.

### 2.7. Microbiological Assessment

The microbiological examination performed on four (previously mentioned) culture mediums (Sheep Blood Agar, Chapman, Lactose Agar, and Sabouraud) provided encouraging results as all the tested samples were negative (0 UFC for each determination) (Figure S1). Out of all, the absence of fungi and aerobic microorganisms was desired in the developed formulations.

### 2.8. The In Vitro AMDR Permeation/Diffusion Test

Until now, no in vitro studies have been conducted. Only ex vivo and in vivo (on animals) studies were conducted where AMDR semisolid formulations were applied on the heart. Considering the application area, it was deemed that this membrane might be the most useful in this case (smaller pore size (0.2 µm)) instead of using the one with the 0.45 µm pore size. The basis for the selection of the synthetic membrane was as follows:

- The membrane size must be large enough to facilitate the permeation of amiodarone hydrochloride and small enough to not allow for the passing of ingredients that are not of interest.
- The membrane composition (to simulate in vivo conditions).

**Figure 9:**
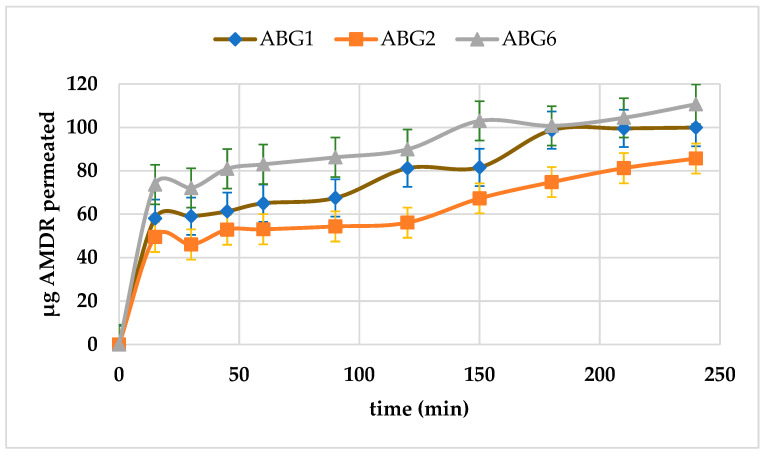
The amount of AMDR that permeated through the synthetic membrane.

Considering the amount of active ingredients that permeated the membrane into the acceptor media, the amounts decreased (at 240 min) in the following order: ABG6 > ABG1 > ABG2. The permeation curves are outlined in Figure 9.

The permeation rate/AMDR flux (240 min) varied between 85.71 ± 6.94 µg/cm^2^ × h (ABG2) and 110.68 ± 6.28 µg/cm^2^ × h (ABG6), a fact that can be correlated with the amount of active ingredient that permeated the synthetic membrane into the acceptor media. JSS ranged between 0.0201 µg/h/cm^2^ (ABG1) and 0.1819 µg/h/cm^2^ (ABG2), and the permeability and diffusion coefficients ranged between 1.34 × 10^−6^ (ABG1) and 12.12 × 10^−6^ (ABG2). Since the synthetic membrane area was close to 1 cm^2^ (A = 0.9993 cm^2^), no differences were recorded between these two coefficients (Table 5).

The differences regarding these parameters can be explained through the different compositions of the bigels. Even though the same lipophilic part and the same emulsifiers in the same amounts were used, the hydrogel composition varied. For ABG1, HGL CMC 5% was used, while for the ABG6 formulation, HGL CMC 3.5% was used. As can be seen, by decreasing the amount of HGL-forming agent, the amount of AMDR released increased. By using HGL-CBP in concentrations of 1% (*w*/*w*), the lowest amounts of API were released, which can be correlated with the CBP-HGL’s increased viscosity.

### 2.9. Future Directions of This Study

The future directions of this study can be divided into different sections, as follows: pharmaceutical product optimization and methodologies that will be used for preclinical trials. In the case of pharmaceutical product optimization, an experimental design where the ratio between the emulsifiers and HGL: OGL and the concentration of the emulsifiers will be varied, using the variables selected in this study as outputs with a focus on the in vitro permeation study. ABG3-ABG5 can also be optimized, considering that in those cases, the OGL amount was too high, a fact that was conducive to phase separation. By reducing the amount of OGL, or by increasing the amount of Carnauba wax and the amount of emulsifiers, more stable formulations might be obtained, as already mentioned in this manuscript. Besides the in vitro study, an ex vivo study on a heart-derived membrane might be useful and helpful for future studies to establish which synthetic membrane can obtain better results than the ones obtained with the ex vivo one. The optimized formulation will be subjected to stability testing at 25 ± 2 °C at 60 ± 5% relative humidity (RH).

Based on the preclinical trials outlined by *Garcia* et al., specific large animals should be selected (Ex. Sprague-Dawley rats), which will undergo anesthesia followed by thoracotomy. Following this procedure, the gels will be administered via a syringe with a predetermined diameter. After 1 month, the rats will be sacrificed, and the heart, lungs, liver, and kidneys will be collected to assay the amount of amiodarone hydrochloride through a previously validated method (HPLC or UV-Vis spectrophotometry) [17].

## 3. Materials and Methods

### 3.1. Materials

Three HGL bases (compositions in Table 6) were obtained, for which the following ingredients were used: Carbopol 940–CBP (Lubrizol, Cleveland, OH, USA), Sodium Carboxymethyl Cellulose–CMC (Qingdao Tianya Chemical Co., Qingdao, China), Glycerol (Chimreactiv SRL, Bucharest, Romania), and Sodium Hydroxide (Vynova, Tessenderlo, Belgium). Using the official method, a preservative solution was prepared beforehand from Methyl Hydroxybenzoate and Propyl Hydroxybenzoate (Fagron, Trikala, Greece).

The first oleogel (OGL) base was prepared using sunflower oil (Argus, Constanța, Romania) and Carnauba wax (Ellemental, Oradea, Romania, origin: Brazil), while the second one was prepared by using almond oil (Fagron, Trikala, Greece), Beeswax (Ellemental, Oradea, Romania, origin: Germany), and Span 60 (Fluka, Buchs, Switzerland). Cosgard (Ellemental, Oradea, Romania, origin: USA) was added to both liquid oils with the composition underscored in Table 7.

To obtain the bigels, the following ingredients and semisolid matrixes were used: Cholesterol (Fagron, Trikala, Greece), Tween 80, Span 80 (PCC Exol SA, Brzeg Dolny, Poland), amiodarone hydrochloride powder (Zhejiang Chemicals Import & Export Corporation, Hangzhou, Zhejiang, China) in a concentration of 1.5% (*w*/*w*), OGL Carnauba, OGL Beeswax, and HGL CMC 3.5%, HGL CMC 5%, and HGL CBP 1%. The codification and composition of the bigels are outlined in Table 8.

A schematic diagram showing the preparation steps for the HGLs, OGLs, and bigels is provided in Figure S2.

### 3.2. Preparation Process

For the bigel preparation, the two components (a hydrophilic part—HGL—and a lipophilic part—OGL) were formulated separately.

#### 3.2.1. Hydrogel Preparation

In the case of CMC gels, glycerol was mixed with water, followed by the gelling agent’s dispersion over the aqueous mixture. In this regard, a magnetic MS-H280-Pro DLAB agitator (DLAB, La Mirada, USA) was used at a speed of 1000 rpm for gel homogenization. In the case of CBP gel, the gelling agent was dispersed over the preservative solution and let into repose for 30 min. After this hydration step, NaOH 10% (*w*/*w*) was added, followed by the addition of the humectant (glycerol). As the final step, to assess the homogeneity of the CBP gel, an automated stirrer was used.

#### 3.2.2. Oleogel Preparation

The preparation method consisted of melting/dissolving the solid ingredients (Carnauba wax in sunflower oil; Beeswax and Span 60 in almond oil) in the heated lipophilic solvent at a maximum temperature of 80 °C and stirring the OGL while cooling to allow for gel formation. Cosgard was added at the end of the preparation stage after the oleogel was cooled at room temperature (22 ± 2 °C). The composition of the two formulations is illustrated in Table 6.

#### 3.2.3. Bigel Preparation

The bigels were obtained by combining equal parts of the previously mentioned bases and by adding the same amount of excipients as follows. For 100 g BGL, 0.5 g of Cholesterol, 3 g of Tween 80, and 2 g of Span 80 were added in the order mentioned for the combined gel bases. Amiodarone hydrochloride powder was incorporated into the mixture in a concentration of 1.5% (*w*/*w*). The ingredients were homogenized in 100 mL containers for 4 min using an automatic mixing device and selecting the 3rd level of speed, corresponding to 1000 rpm. For the first 2 min, equal parts of a HGL and OGL were mixed with the amiodarone powder. Lastly, the other excipients were added and homogenized for the remaining time. The six BGL formulations were transferred to individually labeled boxes (Table 7) and stored at temperatures of 2–8 °C. Additionally, blank formulations were prepared following the same process for the drug content studies excluding the active ingredient from their composition.

### 3.3. Bigel Quality Evaluation

The AMDR BGL formulations were evaluated in terms of stability, spreadability, rheology, API content, textural properties, and microbiological activity. The data collected for penetrometry and drug content were then analyzed statistically using the GraphPad Prism 10 software (Dotmatics, Boston, MA, USA). The experiments were repeated three times for each formulation, and the results were reported as the average ± SD.

#### 3.3.1. Macroscopic Examination and Stability

The separation of the oily and aqueous phases was investigated by studying the changes that emerged in time when stored in cold temperatures (2–8 °C) and through centrifugation. Tubes of 1 mL of bigels were centrifuged for 5 min at a speed of 3000 rpm using the Hettich D-78532 Centrifuge (Hettich, Tuttlingen, Germany).

#### 3.3.2. Spreadability

The consistency examination was based on the gel’s ability to spread when pressed by an external force determined by the application of certain weights of known mass on top of each bigel sample. The determination was performed using the Del Pozo Ojeda-Suñé Arbussá extensometer by weighing 1 g of gel and placing it in the middle of a glass, on top of a millimetric graph paper with concentric circles drawn 10 mm apart. Another glass whose weight ranged between 76.01 and 79.96 g was placed on top of the gel, and after each minute, weights were added in the following order: 50, 100, 200, 300, 400, and 500 g [55]. The diameter of the gel spread was measured, and the surface was calculated using Equation (1):

S = π × r^2^, (1)

where S = gel circle surface (mm^2^); π = 3.14; r = circle’s radius (mm).

#### 3.3.3. Rheology Study

Viscous semisolid systems such as gels require observational rheologic studies due to their ability to flow, which can influence the application and drug delivery results. The test was carried out using the H-cylinders of the Rheotest^®®^ RV viscometer (RHEOTEST Medingen GmbH, Ottendorf-Okrilla, Germany) with 12 shear rates. By increasing the speed until the highest gear, the destructuration of the gel matrix occurred, while restructuration happened by decreasing the speed to the starting point [56]. The monitored parameters are the shear stress (τ) and dynamic viscosity (η).

#### 3.3.4. Drug Assay

The drug content was determined quantitatively through a spectrophotometric method using two wavelengths, 206.9 nm for ABG1 and ABG2 and 242 nm for ABG6. For this, a solution of 40% acetonitrile (ACN) and 60% acetate buffer (pH = 3), previously prepared, was used for obtaining an AMDR stock solution of 100 μg/mL from which the serial dilution of the following concentrations was derived: 1 μg/mL, 2.5 μg/mL, 5 μg/mL, 10 μg/mL, 15 μg/mL, 20 μg/mL, 25 μg/mL, and 50 μg/mL. The calibration curve was established from the absorbances determined with the UV-1800 Shimadzu Spectrophotometer (Mettler Toledo, Columbus, OH, USA) at the two wavelengths.

Additionally, blank bigels with no active ingredient were formulated to perform the baseline correction. To establish the drug content, 1 g of each sample was diluted to 100 mL with the ACN and buffer solution using an automatic MS-H280-Pro DLAB agitator (DLAB, La Mirada, CA, USA) to break the gel matrix, followed by a 1:10 dilution. Before the spectrophotometric measurements, the solutions were filtered using 0.45 μm Millipore, CHROMAFIL Xtra filters (MACHEREY-NAGEL, Düren, Germany).

#### 3.3.5. Textural Analysis

To ensure the quality of the formulations developed, the textural characteristics needed to be investigated. The process was facilitated by the TX-700 Texture Analyser (Lamy rheology, Champagne-au-Mont-d’Or, France). The penetrometry study used a conical attachment, with a 0.05 N force, a top speed of 1 mm/s, and a maximum distance of 200 mm. The Textural Profile Analysis cycle provided insights regarding quantifiable texture attributes by performing two consecutive compressions divided by a resting phase and using the cylindrical attachment. The conditions established for the determination were: 2 mm/s speed, 2 s pause between compressions, sample detection at 0.05 N, and a distance of 15 mm. The values of the textural parameters were calculated based on the graphical representation of the TPA cycle. The peak force of the first compression represents the hardness of the gel, also defined as the gel’s strength. The meaning of cohesiveness refers to the strength of the internal bonds of the matrix. Resilience is centered on the power of the bigel to regain its shape in terms of speed and force, while its elasticity and ability to spring back to the initial height can be translated into springiness. The lowest value of the force registered in the first cycle indicates the adhesion force and positively influences the adhesiveness of the bigels [48,57].

#### 3.3.6. Microbiological Evaluation

Owing to the unique way of applying the developed formulations, it is necessary to ensure their sterility, making them safe for intraoperative usage. An aseptic preparation of the gels was intended, and some culture mediums in aerobic conditions were used to verify the absence of a series of bacteria and fungi. The microbiological study was performed on 5% Sheep Blood Agar, Chapman medium, Lactose Agar, and Sabouraud medium. Most bacteria grow on sheep blood agar; meanwhile, on the Chapman medium, staphylococci grow. Lactose agar was used to differentiate the enterobacteria by using lactose. Sabouraud medium was used to culture fungi; their analysis needed a longer incubation time (48 h) than that for bacteria (24 h).

A total of 10 µL of the bigel probes was transposed on each culture medium with culture swabs and incubated in aerobic conditions at 37 °C for 24 and 48 h.

#### 3.3.7. AMDR In Vitro Diffusion Evaluation Through a Synthetic Membrane

A total of 1 g of each sample (15 mg AMDR/sample) was evaluated for three bigels coded ABG1, ABG2, and ABG6. To assess the amount of AMDR that permeated the membrane, a series of Franz cells (PermeGear, Seelbach, Germany) consisting of a 11.28 mm clear jacket with a flat ground joint, 8 mL acceptor media, pinch clamp, and stir bar were used. The selected acceptor medium was phosphate buffer pH 7.4 containing disodium hydrogen phosphate (Fisher Scientific, Bucharest, Romania), potassium dihydrogen phosphate (Chimopar, Bucharest, Romania), and distilled water, maintained at 37 °C ± 0.1 °C.

During the experiment, at pre-established times, aliquots of 0.8 mL were withdrawn from the Franz cell’s acceptor compartment for 240 min (15, 30, 45, 60, 90, 120, 150, 180, 210, 240 min), which were further replaced with the same volume of fresh phosphate buffer maintained at 37 °C ± 1 °C. To evaluate the permeation process, a synthetic membrane was selected, Express Plus^®^ Membrane (Merck, Cork, Ireland), with a pore size of 0.22 µm, hydrophilic polyethersulfone, and a 9 mm orifice diameter, which required hydration before using it.

The cumulative amounts from the tested formulations were quantified by applying a spectrophotometric UV method at two specific wavelengths (206.9 nm and 242 nm) for the selected active pharmaceutical ingredient.

During the experiment, the following parameters were calculated:

- AMDR flux (J): the quantity that permeated through the membrane divided by the membrane surface multiplied by the time duration (µg/cm^2^ × h) (A = 0.9993 cm^2^).
- Steady state flux: Jss (slope) calculated by utilizing two different methods that produced the same result. The first one consisted of calculating the slope in 180–240 min intervals, and the second employed the LINEST function from Microsoft Excel in the same time frame.
- The permeability coefficient (K_p_ (cm^2^/h)) determined from J and the drug concentration in the donor phase (C_d_ (15,000 µg)) is as follows:

K_p_ = J/C_d_ (2)

- Diffusion coefficient (D)

D = K_p_ × A^2^ (3)

## 4. Conclusions

Due to amiodarone’s lack of solubility and high toxicity profile, bigel formulations were developed and evaluated from a pharmacotechnical point of view to correspond to the desired texture and drug content.

Based on this analysis, three suitable formulations with different gel bases were successfully developed. Although none of them are perfect, all presented strong qualities that sustain this study’s premises. Even if, despite its associated risks, oral amiodarone remains the standard medication for the systemic treatment of arrhythmias due to its proven effectiveness in controlling heart rhythm issues, local administration may be useful in preventing the incidence of postoperative atrial fibrillation. Local atrial drug delivery offers advantages in reducing systemic exposure by avoiding first-pass metabolism and minimizing drug interactions.

## Figures and Tables

**Table 1:** The statistical parameters obtained from spreadability analysis data.

Formulation	Pearson (r) Correlation Coefficient Values	R^2^	Strength	Direction	*p*	Statistical Significance
ABG1	0.9816	0.9634	strong	positive	<0.0001	significant
ABG2	0.9451	0.8931	strong	positive	0.0004	significant
ABG6	0.9828	0.9659	strong	positive	<0.0001	significant

**Table 2:** The values obtained for the spectrophotometric assay in the UV range (n = 10).

Formulation	λ (nm)	C (g%) ± SD
ABG1	206.9	1.43 ± 0.26
ABG2		1.35 ± 0.16
ABG6	242	1.49 ± 0.08

**Table 3:** Penetrometry study results.

No.	Time (s)	ABG1		ABG2		ABG6	
		Force (N)	D (mm)	Force (N)	D (mm)	Force (N)	D (mm)
1	0.008	−0.00758	0.006	−0.00192	0.006	−0.01021	0.005
2	0.532	0.02004	0.519	0.01880	0.508	0.03122	0.513
3	1.057	0.02349	1.042	0.01535	1.033	0.03813	1.038
4	1.581	0.03040	1.567	0.00844	1.556	0.03122	1.561
5	2.105	0.03385	2.091	0.00844	2.081	0.03122	2.086
6	2.637	−0.00068	2.623	0.03951	2.613	0.02086	2.617
7	3.162	0.00623	3.147	0.04642	3.138	0.02432	3.142
8	3.686	0.00623	3.672	0.04297	3.661	0.03467	3.667
9	4.21	0.01313	4.195	0.04642	4.186	0.03813	4.191
10	4.734	0.01313	4.72	0.04297	4.711	0.04158	4.716
11	5.267	0.00277	5.252	0.04642	5.242	0.01051	5.247
12	5.791	0.00623	5.777	0.04642	5.767	0.02086	5.772
13	6.315	0.00277	6.302	0.06023	6.291	0.02432	6.295
14	6.839	−0.00068	6.825	0.06714	6.816	0.02777	6.82
15	7.364	0.01313	7.35	0.07059	7.339	0.03467	7.344
16	7.896	0.00623	7.881	0.04642	7.872	0.02432	7.877
17	8.42	0.00968	8.406	0.05678	8.395	0.02777	8.402
18	8.945	0.00277	8.93	0.06368	8.92	0.02777	8.925
19	9.469	0.00968	9.455	0.07749	9.445	0.03467	9.45
20	10.001	0.04421	9.986	0.07404	9.977	0.04849	9.981
21	10.009	0.03385	9.989	0.10857	9.98	0.07956	9.984

**Table 4:** Textural parameters.

Formulation	Hardness (g)	Cohesiveness	Resilience	Springiness	Adhesion Force (g)
ABG1	75.88	0.628	0.065	1.667	−25.27
ABG2	78.3	0.557	0.051	1.667	−39.99
ABG6	95.76	0.718	0.079	1.666	−21.83

**Table 5:** Permeation/diffusion parameters that were assessed during the experiment.

Code	J (µg/cm^2^ × h) (at 4 h)	JSS (µg/h/cm^2^)	K_p_ × 10^−6^ (cm^−2^ × h^−1^)	D × 10^−6^ (cm^2^/h)
ABG1	99.94 ± 6.94	0.0201	1.34	1.34
ABG2	85.71 ± 6.28	0.1819	12.12	12.13
ABG6	110.68 ± 6.28	0.1658	11.05	11.05

**Table 6:** HGL compositions.

Ingredient	HGL CMC (3.5%)	HGL CMC (5%)	HGL CBP (1%)	
	Amount (% *w*/*w*)			Role
**Sodium Carboxymethyl Cellulose**	3.5	5	-	gelling agent
**Carbopol 940**	-	-	1	
**Glycerol**	3	3	3	humectant
**NaOH 10%**	-	-	3	pH stabilizer
**Preservative solution**	ad 100	ad 100	ad 100	vehicle

**Table 7:** OGL compositions.

Ingredient	OGL Carnauba	OGL Beeswax	
	Amount (% *w*/*w*)		Role
**Carnauba wax**	5	-	gelling agent
**Beeswax**	-	0.2	
**Cosgard**	0.1	0.1	preservative
**Span 60**	-	15	emulsifier/gelling agent
**Sunflower oil**	ad 100		vehicle
**Almond oil**	-	ad 100	

**Table 8:** Codification and composition of BGLs.

Code	Hydrogel (46.5 g)	Oleogel (46.5 g)	Cholesterol (g)	Tween 80 (g)	Span 80 (g)	AMDR (g)
ABG1	HGL CMC (5%)	OGL Beeswax	0.5	3	2	1.5
ABG2	HGL CBP (1%)	OGL Beeswax	0.5	3	2	1.5
ABG3	HGL CMC (5%)	OGL Carnauba	0.5	3	2	1.5
ABG4	HGL CBP (1%)	OGL Carnauba	0.5	3	2	1.5
ABG5	HGL CMC (3.5%)	OGL Carnauba	0.5	3	2	1.5
ABG6	HGL CMC (3.5%)	OGL Beeswax	0.5	3	2	1.5

## Data Availability

All data and materials are available on request from the corresponding author. The data are not publicly available due to ongoing research using a part of the data.

## Supplementary Materials

The following supporting information can be downloaded at https://www.mdpi.com/article/10.3390/ph17111511/s1. Figure S1. Culture mediums: Sheep Blood Agar (**a**), Chapman (**b**), Lactose Agar (**c**), and Sabouraud (**d**). For each medium, the bigels were tested on a Petri dish divided into 4 quadrants. Figure S2. The schematic presentation of the HGL (CMC-Na 5%, CMC-Na 3.5 %, CBP 1%, OGL Carnauba and OGL Beeswax, and bigels (ABG1-ABG6).
