# Supplementary material for: Innovative Epicardial Bigels Containing Amiodarone Hydrochloride: Pharmacotechnical and Analytical Characterization
Source: Pharmaceuticals (Basel). 2024 Nov 9;17(11):1511. doi: 10.3390/ph17111511 (PMC11597466; doi:10.3390/ph17111511)
Supplement: Supplementary file 1 [file pharmaceuticals-17-01511-s001.zip › pharmaceuticals-3289483-supplementary.pdf]

Supplementary material

Innovative Epicardial Bigels Containing Amiodarone Hydrochloride: Pharmacotechnical and Analytical Characterization

Cezara Pintea [1,2,†], Robert-Alexandru Vlad [1,*,†], Paula Antonoaea [1], Emőke Margit Rédai [1], Magdalena Bîrsan [2,3], Enikő-Csilla Barabás [4], Andrei Manea [2,5], Iulia Alexandra Pușcaș [2,6] and Adriana Ciurba [1]

[1] Pharmaceutical Technology and Cosmetology Department, Faculty of Pharmacy, George Emil Palade University of Medicine, Pharmacy, Science and Technology of Targu Mures, 540142 Targu Mures, Romania; cezarapintea@yahoo.com (C.P.); paula.antonoaea@umfst.ro (P.A.); emoke.redai@umfst.ro (E.M.R.); adriana.ciurba@umfst.ro (A.C.)
[2] Medicine and Pharmacy Doctoral School, George Emil Palade University of Medicine, Pharmacy, Science, and Technology of Targu Mures, 540142 Targu Mures, Romania; magdalena.birsan@umfiasi.ro (M.B.); andrei17v1997@gmail.com (A.M.); alexandra.stoica92@yahoo.com (I.A.P.)
[3] Department of Drug Industry and Pharmaceutical Biotechnology, "Grigore T. Popa" University of Medicine and Pharmacy from Iasi, 700115 Iasi, Romania
[4] Department of Laboratory Medicine, Mures, County Hospital, 540136 Targu Mures, Romania; eniko.barabas@umfst.ro
[5] Department of Radiology, Mures, County Emergency Hospital, 540136, Targu Mures, Romania
[6] The Department of Cardiovascular Surgery, Emergency Institute for Cardiovascular Diseases and Transplantation Targu Mures, 540142 Targu Mures, Romania
* Correspondence: robert.vlad@umfst.ro
† These authors contributed equally to this work.

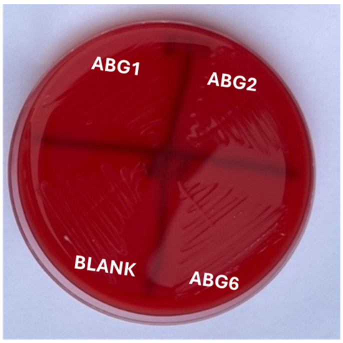

a.

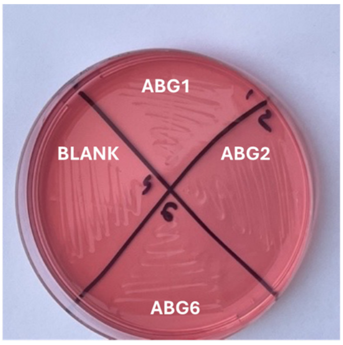

b.

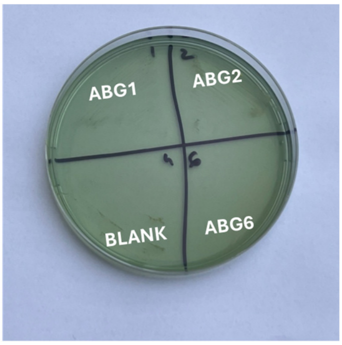

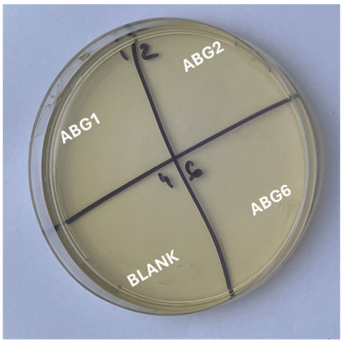

c. d.

Figure S1. Culture mediums: Sheep Blood Agar (a), Chapman (b), Lactose Agar (c), and Sabouraud (d). For each medium, the bigels were tested on a Petri dish divided into 4 quadrants

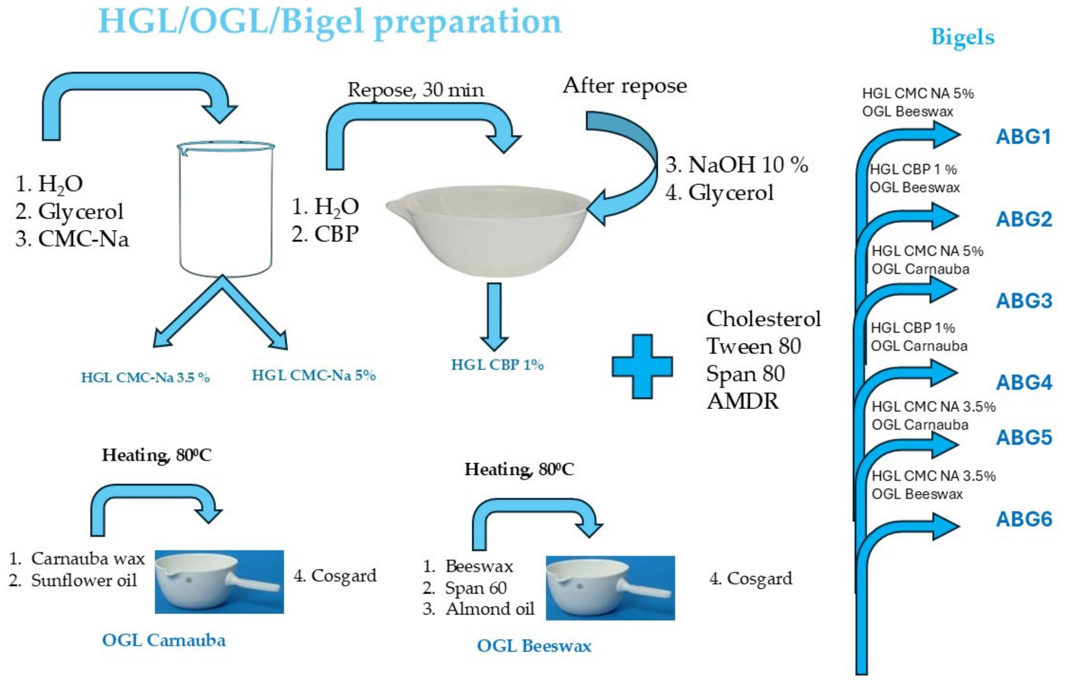

Figure S2. The schematic presentation of the HGL (CMC-Na 5%, CMC-Na 3.5 %, CBP 1%, OGL Carnauba and OGL Beeswax, and bigels (ABG1-ABG6)
